# Multiple Bilateral Incidental Lung Nodules in a Patient with Human Immunodeficiency Virus

**DOI:** 10.7759/cureus.8593

**Published:** 2020-06-13

**Authors:** Muhammad Umar Hayat Khan, Mohammad Uzair Abdul Rauf, Ayesha Mustafa, Richard Silverman

**Affiliations:** 1 Internal Medicine, Yale Waterbury Hospital, Waterbury, USA; 2 Internal Medicine, Akhtar Saeed Medical & Dental College, Lahore, PAK; 3 Pulmonary & Critical Care Medicine, Yale University School of Medicine, Waterbury, USA

**Keywords:** bronchial carcinoid, hiv, lung nodule

## Abstract

Lung nodules are often incidentally discovered on lung imaging and can be solitary, which makes them suspicious for tumors, or multiple, which can be suggestive of an infectious process. A bronchial carcinoid is a rare pulmonary neoplasm, representing 1.2% of all primary pulmonary tumors. We report a case of incidentally discovered multiple lung nodules in an asymptomatic human immunodeficiency virus (HIV) patient, which turned out to be a tumor, necessitating the need for keeping a broad differential, a high degree of clinical suspicion, and long-term follow-up for the optimal management of the patient.

## Introduction

Bronchial carcinoids are low-grade, malignant neuroendocrine tumors ranging from low-grade typical to more aggressive atypical carcinoids. They are believed to arise from Kulchitsky's cells of the bronchial epithelium. It is unclear whether there is an association of bronchial carcinoid with human immunodeficiency virus (HIV) infections. However, gastrointestinal carcinoids are more common than pulmonary carcinoids in HIV-infected patients [1]. We report an interesting case of a middle-aged woman with an HIV infection who was found to have multiple, bilateral, incidental lung nodules, which were further diagnosed to be a carcinoid tumor.

## Case presentation

A 54-year-old Hispanic female with a past medical history of HIV and cervical carcinoma in situ presented to the emergency department with a complaint of left-sided flank pain associated with dysuria, hematuria, and fever. Her most recent cluster of differentiation 4 (CD4) count was 1301 cells/microliter and the HIV viral load was less than 20 copies/milliliter. She did not have a history of asthma or chronic obstructive pulmonary disease (COPD). She is a housewife, who quit smoking about 15 years ago and did not drink alcohol or take illicit drugs.

She was afebrile, tachycardic, with left costovertebral angle tenderness and positive bowel sounds. She had leukocytosis with a left shift, elevated creatinine from the baseline, and urinalysis suggestive of bacteriuria. The computed tomography (CT) scan of the abdomen showed bilateral hydronephrosis, non-obstructing right renal calculi, and possible calculus in the right ureter with perinephric fat stranding suggestive of pyelonephritis. Her urine cultures grew Escherichia coli, and she was treated with seven days of levofloxacin based on culture sensitivities. Urology was consulted, and a ureteral stent was placed while in the hospital with close outpatient follow-up.

**Figure 1:**
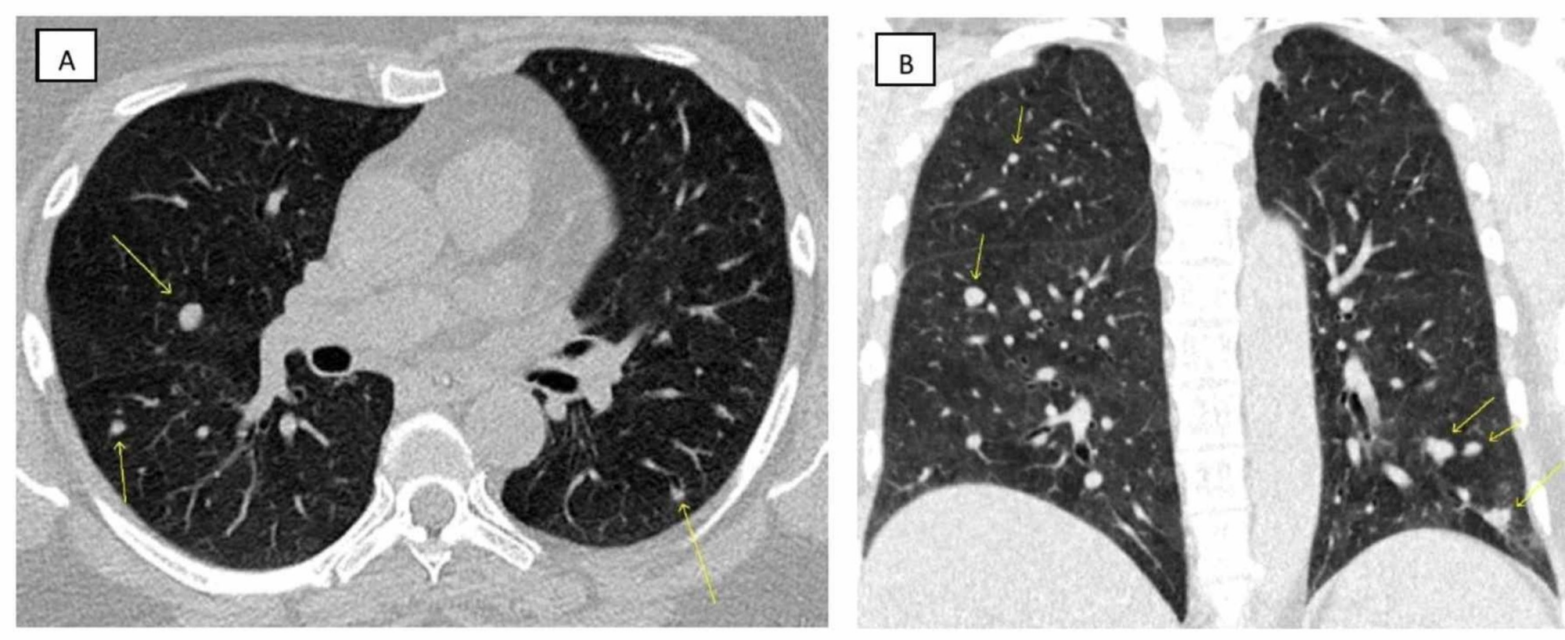
Chest computed tomography A, An axial window of the lung shows a rounded, about 7 mm, prominent nodule in the right lower lobe along with a few other nodules in the right and left lung (arrow); B, A coronal section of the lung shows the presence of multiple lung nodules (arrow)

The CT scan of the abdomen also incidentally showed bilateral lower lobe lung nodules. A dedicated CT scan of the chest also confirmed the presence of bilateral lung nodules with the largest measuring up to 7 mm in the right lower lobe (Figure 1). There was no history of cough, hemoptysis, or weight loss. The patient subsequently had a positron emission tomography (PET) scan as an outpatient, which was negative for a hypermetabolic focus in the lungs and lymph nodes.

**Figure 2:**
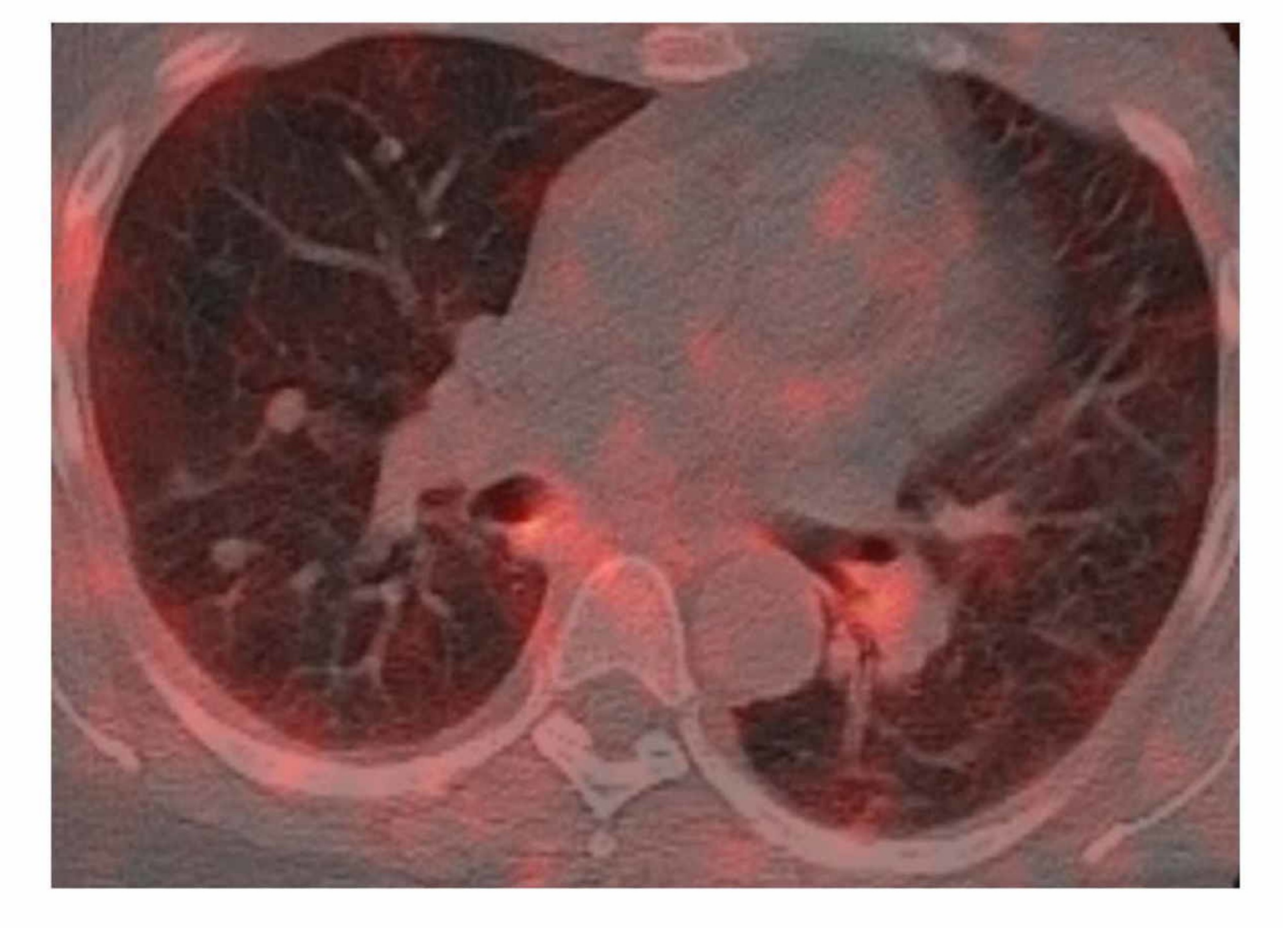
Carcinoid tumor Positron emission tomography (PET) scan is negative for hypermetabolic activity in the lungs

She underwent a repeat chest CT scan for two years, with interval stability of most of the bigger nodules but an enlargement of the few smaller nodules. She had another PET scan from the skull base to mid-thigh, which was negative for a hypermetabolic focus in the lungs and lymph nodes (Figure 2).

**Figure 3:**
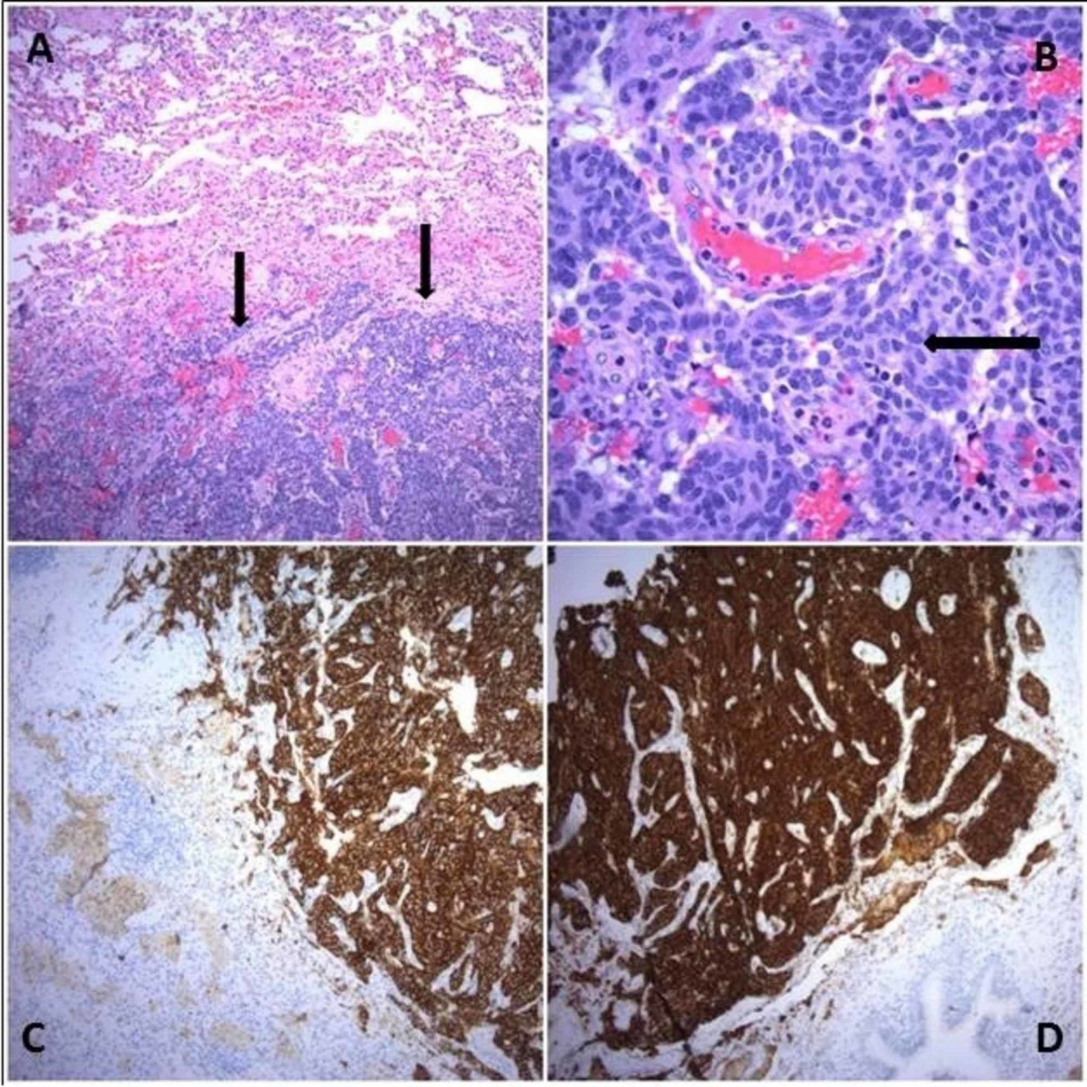
Carcinoid tumor (A) Tumor cells with salt and pepper chromatin pattern (hematoxylin & eosin (H&E) x 200); (B) The cell nuclei are ovoid, dense, and uniform {Arrow shows one of the many nuclei} (H&E x 400) The tumor cells are strongly positive for synaptophysin (C) and chromogranin (D) (immunohistochemistry: x 200).

She was scheduled for a thoracoscopic lung biopsy from the right lower lobe. The biopsy specimen was sent for acid-fast/smear, fungal culture, gram stain, and bacterial culture, which were all negative. The pathology report showed multifocal, uniform, ovoid neuroendocrine-type cells, with typical "salt and pepper" chromatin patterns measuring 0.6 cm in the maximum dimension. Immunohistochemical staining showed strong positivity for chromogranin and synaptophysin with weak mitotic activity (Figure 3). These findings are consistent with a typical carcinoid.

Due to the location and burden of the nodules, curative resection (lobectomy or pneumonectomy) could not be pursued in our patient. No chemotherapy or radiotherapy was pursued due to the typical and non-aggressive nature of the carcinoid and since the patient was asymptomatic. We will continue to follow up with the patient, closely monitoring her for the development of any symptoms, and conduct interval chest imaging every three years to ensure the stability of the lung nodules.

## Discussion

A bronchial carcinoid is an uncommon and indolent pulmonary neoplasm, representing 1.2% of all primary pulmonary tumors [2]. It usually occurs in the fifth decade of life and primarily involves the gastrointestinal system, lungs, kidneys, and ovaries. Patients with a bronchial carcinoid usually present with hemoptysis, sputum production, fever, flushing, diarrhea, and wheezing [3]. The diagnosis of a bronchial carcinoid in our patient was very challenging due to the lack of clinical suspicion and its rare occurrence. Our initial thought was a pulmonary infection or lung cancer, which are the common causes of multiple pulmonary nodules in HIV patients. Lungs are one of the main organs involved in diseases related to HIV and during the course of illness, almost 70% of patients suffer from at least one respiratory complication [4]. In a retrospective study performed at San Francisco General Hospital, in 242 patients with HIV, 36% of patients had one or more pulmonary nodules on CT chest. Opportunistic infections were the leading cause, followed by bacterial pneumonia, tuberculosis, Kaposi’s sarcoma, lymphoma, and lung cancer [5]. Our case emphasizes the importance of keeping a broad differential in HIV-infected patients with multiple pulmonary nodules. It is also important to be aware of the fact that due to the introduction of antiretroviral therapy and its widespread use, the incidence of non-AIDS defining cancers is on the rise.

The paucity in data regarding bronchial carcinoids in HIV-infected patients suggests that it is rare. We found two case reports during our literature search where a bronchial carcinoid was found in a patient with HIV. One of them was a middle-aged gentleman with HIV and hepatitis C, who presented with shortness of breath, right-sided pleuritic chest pain, and productive cough and was found to have an 8 mm obstructing lesion of the superior segment of the right lower lobe. Biopsy of the lesion showed an atypical carcinoid tumor [6]. However, the other case was of a 60-year-old male patient with HIV who presented with shortness of breath, palpitations, and weight loss and was found to have a left lower lobe endobronchial mass. It was found to be a typical bronchial carcinoid on brush cytology smears [7]. On the contrary, our patient was asymptomatic and was incidentally found to have multiple bilateral lung nodules and, subsequently, a biopsy-proven, typical bronchial carcinoid.

The radiologic features of bronchial carcinoids depend on tumor location, and these features are the same for both typical and atypical carcinoids. Almost 80% of bronchial carcinoids arise centrally, either in the main lobar or segmental bronchi [3]. They appear as hilar or perihilar masses, often demonstrating radiologic evidence of an endobronchial component and can cause hemoptysis or recurrent bronchial obstruction. On the other hand, about 20% of bronchial carcinoids present in the lung periphery and manifest as a solitary pulmonary nodule [3]. Multifocal pulmonary carcinoids have rarely been reported. In contrast, our patient had a rare presentation, with multiple small bilateral bronchial carcinoids, and the largest nodule measuring up to 7 mm. It is unclear whether this unique presentation is related to or due to an HIV infection or not.

Serum chromogranin A (CgA) has low specificity and is not used for screening purposes. A CT scan is the gold standard for diagnosing a pulmonary carcinoid. Somatostatin receptor scintigraphy (octreoscan) helps localize tumor sites. A pathological examination is important for the correct classification of carcinoids.

The five-year survival rate for a typical pulmonary carcinoid is about 87%-100% compared to a worse prognosis with the atypical form, which is about 35%-56%. The symptoms of carcinoid syndrome are managed with a somatostatin analog. A metastatic and aggressive carcinoid tumor is treated with antineoplastic and cytotoxic medication. Resection is essentially curable for a localized carcinoid lesion. However, our patient had multiple, bilateral, tiny bronchial carcinoids not amenable to resection and given their typical nature and no symptoms, the patient is not a candidate for chemotherapy. European Neuroendocrine Tumor Society (ENETS) recommends conventional cross-sectional imaging at three and six months and then every 12 months for typical carcinoids, together with a measurement of CgA for the first two years, followed by an annual chest radiograph and biochemistry profile and a CT every three years long-term [8].

## Conclusions

A bronchial carcinoid is an uncommon pulmonary neoplasm. Its association with an HIV infection is unclear due to the paucity of data. Our case emphasizes the importance of keeping a broad differential, a high degree of clinical suspicion, and long-term follow-up for the optimal management of HIV-infected patients with multiple pulmonary nodules.
